# Exploring the nurse-patient relationship in caring for the health priorities of older adults: qualitative study

**DOI:** 10.1186/s12912-024-02099-1

**Published:** 2024-07-15

**Authors:** Mostafa Shaban, Huda Hamdy Mohammed, Fatma Gomaa Mohamed Amer, Marwa Mamdouh shaban, Hassanat Ramadan Abdel-Aziz, Ateya Megahed Ibrahim

**Affiliations:** 1 Faculty of Nursing, Cairo University, Cairo, Egypt; 2 Community Health Nursing, Faculty of Nursing, Ain Shams University, Cairo, Egypt; 3 Community Health Nursing, Faculty of Nursing- Cairo University, Cairo, Egypt; 4 Department of Nursing, College of Applied Medical Sciences in Al-Kharj, Prince Sattam Bin Abdulaziz University, Al-Kharj, 11942 Saudi Arabia; 5 Gerontological Nursing Department, Faculty of Nursing, Zagazig University, Zagazig, Egypt; 6 College of Nursing, Prince Sattam bin Abdulaziz University, Al-Kharj, 11942 Saudi Arabia; 7 Family and Community Health Nursing Department, Faculty of Nursing, Port Said University, Port Said, Egypt

**Keywords:** Person-centered care, Nurse-patient relationship, Older adults, Outpatient settings, Qualitative research

## Abstract

**Background:**

Person-centered care (PCC) is critical in addressing the diverse health priorities of older adults. Nurses play a pivotal role in implementing PCC, yet the nuances of the nurse-patient relationship in outpatient settings remain underexplored. This study aimed to gain insights into nurses’ experiences, challenges, and strategies in caring for older adults through the lens of PCC.

**Methods:**

A qualitative descriptive design was employed, involving semi-structured interviews with 12 registered nurses from outpatient clinics serving older adults. Thematic analysis was conducted following the principles of trustworthiness and credibility.

**Results:**

Five main themes emerged: (1)Understanding and Implementing Person-Centered Care (PCC) (2) Experiences in Older Adult Care, highlighting the significance of trust-building, adapting care approaches, interdisciplinary collaboration, and emotional rewards; (3) Challenges in Care Delivery, including resource constraints, navigating family dynamics, keeping up with medical advances, and emotional strain; (4) Impact on Care Quality, encompassing consistency in care, patient satisfaction, professional development, and ethical considerations; and (5) Coping Strategies, such as peer support, work-life balance, reflective practice, and resilience building.

**Conclusions:**

The study underscores the complexities and rewards of the nurse-patient relationship in caring for older adults in outpatient settings. Nurses face formidable challenges but employ various coping strategies to maintain high-quality, person-centered care. Findings have implications for nursing practice, education, policy, and future research, emphasizing the need for supportive environments, continuous professional development, and recognition of the critical role nurses play in addressing the health priorities of the aging population.

**Supplementary Information:**

The online version contains supplementary material available at 10.1186/s12912-024-02099-1.

## Introduction

The concept of Person-Centered Care (PCC) is fundamental to enhancing the quality of healthcare, particularly in outpatient settings where nurses interact frequently with older adults [1]. This approach not only focuses on the medical needs of patients but also emphasizes understanding and respecting their personal values and preferences. However, effectively implementing PCC requires a nuanced understanding of the dynamics between nurses and patients, which is often overlooked in research focused primarily on clinical outcomes [2].

Person-Centered Care (PCC) represents a paradigm shift in healthcare delivery, where the focus is directed toward the holistic understanding of patients as individuals with unique needs, preferences, and values [3]. Defined by the Health Foundation as “an approach to care that consciously adopts the perspectives of individuals, families, and communities to ensure that patient values guide all decisions” [4], PCC underscores the importance of integrating the patient’s voice into the care process [5]. This approach is particularly pivotal in geriatric nursing, where the complexity of care needs demands a comprehensive understanding that transcends mere clinical interventions [6]. In caring for older adults, PCC emphasizes the significance of addressing psychological, social, and spiritual well-being alongside physical health, thus advocating for a care model that is as diverse as the patient population it serves [7].

Nurses, as the primary point of contact for patients, are uniquely positioned to drive the adoption of PCC principles [8]. Their frontline role enables them to build therapeutic relationships that are essential for effective PCC, allowing for a deeper understanding of patients’ needs and preferences [9]. This shift towards PCC in nursing has been supported by evidence linking PCC to better patient outcomes, including reduced hospitalization rates and improved chronic condition management [10]. Moreover, the emphasis on PCC has also led to greater job satisfaction among nurses, as it aligns with a more meaningful and rewarding approach to patient care [11].

Person-Centered Care (PCC) takes on a critical role in the healthcare of older adults, a demographic uniquely impacted by a spectrum of health challenges such as chronic diseases, multimorbidity, and the natural process of aging which can lead to functional decline [12]. This necessitates a care approach that transcends traditional medical treatment to embrace a more holistic view, recognizing the importance of psychological, social, and spiritual well-being alongside physical health [13]. PCC, in this context, demands a shift from a one-size-fits-all model to a more nuanced and individualized care strategy [14]. This approach not only aims to manage the medical aspects of care but also to address the broader dimensions of health, ensuring that the care plans for older adults are tailored to their specific needs, preferences, and life circumstances [15].

The nurse-patient relationship is pivotal in the effective delivery of PCC to older adults [16]. It is through this relationship that nurses can truly understand the unique needs and preferences of each older adult, fostering a foundation of trust and mutual respect [17]. This connection is built on the principle of viewing each patient not just as a recipient of care but as a partner in the care process [18]. Nurses who practice PCC are committed to listening to the stories of older adults, understanding their life experiences, values, and preferences, and integrating this knowledge into the planning and delivery of care [19]. This relationship-centric approach empowers older adults, actively involving them in making informed decisions about their care and treatment options, thereby supporting their autonomy and independence [20].

Addressing the health priorities of older adults through Person-Centered Care (PCC) and the nurse-patient relationship requires an understanding of the complex and varied needs of this population [21]. Older adults face a wide range of health challenges, from managing chronic conditions like diabetes and hypertension to addressing mental health issues such as depression, anxiety, and dementia [22]. Additionally, promoting healthy aging—which includes maintaining physical, social, and mental well-being—is crucial [23]. These diverse needs demand a holistic approach to care, one that goes beyond treating physical symptoms to address the full spectrum of factors affecting an individual’s health [24].

PCC plays a pivotal role in meeting these health priorities by focusing on the unique needs and preferences of each older adult [25]. This approach relies on a multidisciplinary team of healthcare professionals, with nurses often at the forefront, working collaboratively to provide comprehensive care [26]. Such an approach not only improves health outcomes but also enhances the quality of life for older adults, making it a key strategy in the promotion of healthy aging [27].

The emphasis on Person-Centered Care (PCC) has highlighted the necessity of understanding the unique environments in which healthcare is delivered [28]. While much of the literature has focused on inpatient care, outpatient settings offer a distinct landscape for the implementation of PCC [29]. Unlike inpatient settings, where care is intensive and environments are controlled, outpatient settings present unique challenges and opportunities for PCC [30]. These include logistical constraints, the necessity for streamlined communication, and the importance of integrating care with patients’ daily lives. Understanding these differences is crucial for tailoring PCC approaches to meet the specific needs of older adults in outpatient care [31]. Furthermore, outpatient care settings often require a more collaborative approach to healthcare, involving a network of primary care providers, specialists, and community resources to support the patient’s health outside of the traditional hospital environment [32]. This necessitates a distinct approach to Person-Centered Care (PCC), one that emphasizes continuity of care, patient education, and self-management support. Understanding and addressing these unique challenges and opportunities are vital for developing and implementing effective PCC strategies tailored to the needs of older adults in outpatient settings. Additionally, it is crucial that providers listen carefully to the patients’ wishes and preferences, ensuring that their voices are central to the care planning process. This patient-centered listening is fundamental to creating care plans that truly reflect the individual needs and desires of older adults, fostering a more collaborative and respectful healthcare environment [31, 33, 34] .

Despite the myriad challenges, the opportunities for enhancing care through Person-Centered Care (PCC) are significant [35]. Leveraging technology and evidence-based practices, nurses are uniquely positioned to transform the care landscape for older adults. The nurse-patient relationship, rooted in mutual respect and understanding, is central to this transformation, enabling nurses to advocate for and implement care strategies that truly reflect the preferences and needs of older adults [36]. PCC not only addresses diverse health priorities but also establishes a new standard for compassionate, holistic care in nursing practice, representing a significant step forward in the pursuit of optimal health and well-being for the aging population [15].

### Aim of the study

The aim of this qualitative study was to explore the intricate dynamics of the nurse-patient relationship in the context of caring for older adults in outpatient settings, with a specific emphasis on person-centered care (PCC) practices. In our study, we explored the concept of PCC as experienced and interpreted by outpatient nurses working with older adults. Rather than imposing a rigid definition, we sought to understand PCC through the lens of the nurses, allowing their experiences and interpretations to illuminate the multifaceted nature of PCC in practice.

**Research questions**

1. What are the experiences, perceptions, and challenges faced by nurses in building and maintaining nurse-patient relationships while delivering person-centered care to older adults in outpatient settings?
2. What strategies and coping mechanisms do nurses employ to navigate the complexities of caring for older adults, promote their well-being, and maintain high standards of person-centered care in outpatient clinics?

## Methods

### Study design

This study employed a qualitative descriptive design, deeply rooted in the epistemological frameworks of naturalism and constructivism. Naturalism posits that realities are multiple and subjective, while constructivism emphasizes the interaction between the researcher and the subject in shaping the findings [37, 38]. These frameworks were chosen for their ability to capture the complex, lived experiences and nuanced interactions between healthcare professionals and patients within the scope of Person-Centered Care (PCC) [39].

To ensure methodological rigor and transparency, our approach adhered closely to the Standards for Reporting Qualitative Research (SRQR) guidelines. This adherence facilitated a systematic and reflective inquiry into the nurse-patient relationship, allowing for detailed exploration and credible documentation of emergent themes that authentically represent participants’ experiences [40]. By integrating these epistemological principles, we aimed to illuminate the subjective and often tacit knowledge that informs the practice of PCC in nursing, particularly in outpatient care settings [40]. . This methodological framework was chosen for its ability to capture the complexity and variability of real-world nursing practices, providing valuable insights into the delivery of person-centered care.

### Study setting

The study was conducted in outpatient clinics affiliated to Zagazig university educational hospitals. These clinics were selected for their high volume of older adult patients and the comprehensive nature of services they provide, including management of chronic conditions, acute care, and mental health services. This setting was chosen to capture a broad spectrum of nurse-patient interactions and the varied health priorities of older adults in environments where they routinely seek care. The outpatient clinic environment offers a unique context for examining the nurse-patient relationship, as it encompasses both episodic and ongoing care scenarios, providing a rich backdrop for exploring how nurses navigate and prioritize the health needs of older adults in a setting characterized by both continuity and immediacy of care.

### Participants

A purposeful sampling technique was utilized to recruit 12 registered nurses from outpatient clinics for this study. The participants were drawn from a diverse range of specialties within the outpatient setting to ensure a comprehensive understanding of the nurse-patient relationship in caring for older adults. Among the participants, 9 were females and 3 were males, reflecting the gender distribution commonly observed in the nursing profession. The ages of the participants ranged from 28 to 55 years, providing a broad perspective across different stages of nursing careers. In terms of educational background, the participants varied from holding diplomas in nursing to advanced degrees, including bachelor’s and master’s degrees in nursing. This diversity in education levels contributed to a broad range of insights into the practice of person-centered care, reflecting different levels of training and perspectives on patient care.—this seems redundant or could be synthesized into the first paragraph. The study explicitly focused on registered nurses who had direct patient contact in outpatient settings, excluding those in administrative roles without regular patient interactions or those working primarily in inpatient settings. This focus ensured that the study captured the nuances of the nurse-patient relationship in the specific context of outpatient care for older adults.—combine with the above paragraph.

The selection criteria ensured that each nurse had a minimum of 5 years in the nursing field, with at least 3 years specifically dedicated to working in outpatient clinics that serve a significant number of older adult patients. This criterion was set to capture insights from nurses who have had substantial interaction with the older adult population and could provide detailed accounts of their experiences and strategies in managing the health priorities of this group. The nurses represented various specialties critical to the comprehensive care of older adults, including general medicine, chronic condition management, geriatric care, and mental health services. This variety allowed for a richer exploration of the nurse-patient relationship across different health care needs of older adults.

### Development of interview guide

The development of our interview guide was a meticulous process influenced by established frameworks and prior studies that explored the dynamics of nurse-patient relationships within the framework of Person-Centered Care (PCC). Specifically, the guide drew upon the foundational work by Kitwood (1997) on person-centered approaches in dementia care, which emphasizes understanding the individual’s perspective and adapting care practices accordingly [25]. Additionally, we incorporated insights from McCormack’s (2004) study on person-centeredness in nursing homes, which provided valuable structures for questioning techniques that elicit detailed narratives about care experiences [41].

To tailor these frameworks to our specific research context—outpatient clinics serving older adults—we conducted a preliminary literature review to identify gaps in existing studies, particularly focusing on the outpatient setting’s unique challenges and opportunities. Based on this review, our team crafted questions that were designed to probe deeper into the specific experiences of nurses in these settings. Questions were formulated to explore themes such as the implementation of PCC principles, the challenges faced in establishing trust with older adults, and strategies for personalized care planning.

The draft guide was then reviewed by a panel of experts in qualitative research and geriatric nursing. Their feedback helped refine the questions to ensure they were open-ended yet specific enough to elicit detailed and relevant responses. The final interview guide was piloted with a small group of nurses from a local outpatient clinic to test the clarity and effectiveness of the questions. Adjustments were made based on this pilot to ensure that the questions were comprehensible and effectively encouraged rich, informative dialogue.

### Data collection

Data collection was conducted through face-to-face, in-depth interviews using a semi-structured interview guide specifically designed for this study (referenced in Table S1), which was meticulously developed and reviewed by language experts and co-authors to ensure comprehensiveness and relevance. Each interview was audio-recorded and conducted in a private room within the outpatient clinic, lasting approximately 45 min to provide a conducive environment for open sharing.

Interviews were conducted in both English and Arabic, depending on participants’ preferences, with bilingual experts ensuring accurate translation and integrity of the data. The translation process involved a rigorous two-step verification to capture linguistic nuances and healthcare terminologies, ensuring that translated materials accurately reflected original statements.

In addition to interviews, data collection included observational notes and document reviews. Notes captured immediate reflections and non-verbal cues, enriching the verbal data. Observations within outpatient settings provided insights into the application of PCC, focusing on non-verbal communications and environmental interactions. All observational and interview data were systematically analyzed alongside reviewed documents, such as care plans and clinic policies, to ensure a comprehensive understanding of PCC practices and their institutional alignment. Observations were conducted to capture the non-verbal interactions and environmental contexts of nurse-patient interactions. Specific behaviors, communication patterns, and care delivery practices were noted. Observations focused on how nurses applied PCC principles in real-time patient interactions.

### Credibility of the study

In this study, the credibility of the findings was ensured through data triangulation, integrating multiple sources and methods to provide a comprehensive understanding of Person-Centered Care (PCC) practices and nurse-patient relationships. The three primary data sources used for triangulation were:

1. **Semi-structured interviews**
  **Core Data Source**: In-depth, semi-structured interviews with 12 registered nurses from outpatient clinics were conducted. These interviews offered rich qualitative insights into the nurses’ experiences, perceptions, and strategies in delivering PCC to older adults.
  **Purpose**: The interviews were designed to explore how nurses implement PCC, the challenges they encounter, and the coping mechanisms they employ. This data formed the foundation for understanding the dynamics of the nurse-patient relationship.
2. **Document analysis**
  **Reviewed Documents**: Documents such as patient care plans, clinic policies, and guidelines for implementing PCC were reviewed. These documents provided institutional context and procedural frameworks for PCC practices.
  **Purpose**: Document analysis served to cross-verify the qualitative data obtained from interviews. It offered additional context and perspective, highlighting how institutional policies support or constrain PCC, thereby complementing and contextualizing the interview findings.
3. **Observational Data**
  **Observed Interactions**: Observations focused on real-time interactions between nurses and older adult patients in outpatient settings. These observations captured non-verbal communication, practical applications of PCC principles, and immediate responses to patient needs.
  **Purpose**: Observational data added an experiential dimension to the study, enriching the understanding of how PCC is practiced in real-world settings. It provided concrete examples of nurse-patient interactions, validating the narratives obtained from interviews and the procedural insights from document analysis.

To enhance the credibility and validity of the findings, several methodological strategies were employed:

- **Triangulation**: Data from interviews, document analysis, and observations were systematically cross-verified to ensure consistency and robustness. This process involved comparing and contrasting information from each source to build a comprehensive and reliable understanding of PCC practices.
- **Collaborative Analysis**: The primary researcher conducted data collection through interviews and observations. Co-researchers participated in data analysis, contributing diverse perspectives and insights. This collaborative approach facilitated a more nuanced interpretation of the data and helped mitigate potential biases.
- **Peer Debriefing**: Regular peer debriefing sessions were conducted with the research team to review and validate the emerging themes and findings. This iterative process allowed for critical examination and refinement of the analysis, enhancing the study’s credibility.

### Data analysis

Data analysis for this study followed a thematic approach, based on the framework proposed by Braun & Clarke (2006) [42], involving six key phases: (1) Familiarization with the data, (2) Generating initial codes, (3) Searching for themes, (4) Reviewing themes, (5) Defining and naming themes, and (6) Producing the report as shown in Table 1.

1. **Familiarization with the data**
  **Transcription and Initial Reading**: Verbatim transcriptions of the semi-structured interviews were created by two trained researchers, capturing the exact responses of the participants. Interviews conducted in Arabic were simultaneously translated into English by bilingual investigators. To ensure the accuracy of the translations, an independent research assistant cross-verified selected English transcripts with the original Arabic recordings.
  **Immersion**: The research team engaged in multiple readings of the transcripts to become thoroughly familiar with the content, noting initial impressions and significant patterns.
2. **Generating initial codes**
  **Coding Process**: Initial codes were generated systematically across the entire dataset, focusing on meaningful segments of text that captured recurring themes and patterns. These codes represented key aspects of the participants’ experiences and perceptions regarding Person-Centered Care (PCC) and the nurse-patient relationship.
  **Collaborative Effort**: The coding process was iterative and involved discussions among the research team to refine and validate the initial codes. This collaboration helped ensure that the codes accurately reflected the data.
3. **Searching for themes**
  **Organizing Codes**: Codes were then organized into potential themes by grouping related codes together. This phase involved looking for broader patterns and relationships among the codes to form coherent thematic categories.
  **Initial Theme Development**: The team identified several preliminary themes that encapsulated significant aspects of the data. These themes were discussed and refined to ensure they were comprehensive and reflective of the participants’ experiences.
4. **Reviewing themes**
  **Theme Refinement**: The identified themes were reviewed and refined by the research team to ensure they accurately represented the data. This process involved checking if the themes worked in relation to the coded extracts and the entire dataset.
  **Consensus-Building**: The team engaged in collaborative discussions to resolve any discrepancies in theme identification and refinement, fostering a consensus-driven approach.
5. **Defining and naming themes**
  **Finalizing Themes**: Each theme was defined and named to clearly convey its essence and relevance to the research questions. Detailed definitions and descriptions were developed for each theme, highlighting the core concepts and insights derived from the data.
  **Sub-Themes**: Where applicable, sub-themes were identified to capture more specific aspects of the broader themes, providing a nuanced understanding of the data.
6. **Producing the report**
  **Reporting**: The final phase involved producing a comprehensive report of the findings, integrating the themes into a coherent narrative that addressed the research objectives. The report included direct quotes from participants to illustrate and support the themes, ensuring that the voices of the nurses were authentically represented.

**Table 1:** Examples of how codes were clustered into subthemes

Subtheme	Initial Codes	Description
Defining Person-Centered Care	Holistic approach mention, Patient values emphasis	This subtheme encompasses nurses’ descriptions that formed a collective definition of PCC, emphasizing holistic, values-based care.
Providing Person-Centered Care	Individual preference accommodation, Care plan collaboration	This subtheme illustrates the strategies and approaches employed by nurses to operationalize PCC, highlighting how they engage patients in their care and ensure treatment plans reflect personal preferences and life contexts.
Barriers to Person-Centered Care	Time constraint frustration, Resource limitation	Focuses on external factors that challenge the delivery of PCC, bringing attention to systemic issues impacting the feasibility of fully implementing PCC practices.

### Ethical consideration

This study received approval from the Institutional Review Board (IRB) of the Faculty of Nursing, Zagazig University, Egypt, with the reference number ID: ZU.NUR.REC#:085 in November 2023. Permissions were also secured from the outpatient clinics where the research was conducted. Informed consent was a priority. Participants received detailed explanations about the study’s objectives, methods, and their rights, including the right to withdraw at any time without any repercussions. Specifically, for the physical observations of care processes, participants were informed that their interactions might be observed, noting how these observations would be used solely for research purposes. They were assured that no personally identifiable information would be recorded during these observations. To ensure confidentiality and anonymity, each participant was assigned a unique identifier. All personal information and observational data were securely stored and accessible only to the research team. Rigorous measures were implemented to safeguard this data from unauthorized access. Additionally, we took precautions to mitigate any potential psychological discomfort during interviews and observations by providing immediate access to support services and maintaining a respectful and non-intrusive observation process.

## Results

Can the results be summarized more through the use of a table and then only explaining the overarching themes that incorporated the sub-themes?

The characteristics of participants (Table 2) demonstrate the range in age from 28 to 58 years, and showcasing a broad spectrum of experience that is likely to influence their caregiving approaches and perspectives. With a gender distribution predominantly female, reflecting common nursing workforce demographics, the inclusion of male nurses ensures a more comprehensive view of nursing care dynamics. Educational backgrounds vary from Diplomas to master’s Degrees, indicating a diverse set of academic preparations that could impact person-centered care practices. Experience levels in outpatient settings range widely from 5 to 25 years, suggesting that the study benefits from a mix of seasoned insights and fresh perspectives. This diversity among participants is crucial for a nuanced understanding of the complexities surrounding the nurse-patient relationship and the delivery of care to older adults in outpatient settings.

**Table 2:** Characteristics of Nurse Participants-not sure indiivudal detail is informative vs. an aggregate summary

Participant	Age (Years)	Gender	Education Level	Years of Nursing Experience in Outpatient Settings
N1	34	Female	Bachelor’s Degree	10
N2	46	Female	Master’s Degree	15
N3	39	Male	Bachelor’s Degree	8
N4	58	Female	Advanced Diploma	25
N5	30	Female	Bachelor’s Degree	5
N6	58	Female	Diploma	25
N7	44	Male	Bachelor’s Degree	12
N8	49	Female	Diploma	18
N9	37	Male	Bachelor’s Degree	9
N10	41	Female	Bachelor’s Degree	16
N11	28	Female	Bachelor’s Degree	7
N12	53	Female	Diploma	22

In the exploration of nurse-patient relationships within the context of caring for older adults in outpatient settings, our study delved into the intricate dynamics that shape these interactions. Through qualitative analysis, we identified key themes and subthemes that capture the essence of nurses’ experiences, the challenges they encounter, and the strategies they employ to navigate these challenges effectively (Table S2). The findings, distilled from in-depth interviews and observational data, shed light on the multifaceted nature of providing person-centered care to older adults. These insights are crucial for understanding how nurses adapt to and overcome the complexities of their roles, ensuring that the care they provide aligns with the health priorities and personal preferences of older adults. The thematic results, summarized in the subsequent table, offer a comprehensive overview of the critical aspects of nursing care in outpatient settings, highlighting the importance of trust-building, interdisciplinary collaboration, and the emotional and professional growth of nurses.

### Understanding and implementing person-centered care (PCC)

#### Defining person-centered care

Nurses in our study articulated PCC as a multifaceted approach that prioritizes understanding and integrating the unique preferences, values, and needs of each patient into their care. For instance, Participant N1 described PCC as “seeing the person beyond their diagnosis, understanding their life outside the hospital walls, and weaving that understanding into the fabric of our care.” This definition underscores the shift from a disease-centric to a holistic view of care. Participant N4 highlighted the adaptability required in PCC, stating, “It’s about customizing our approaches to align with each patient’s life context, ensuring care is both respectful and responsive.” These definitions collectively paint PCC as an approach that encompasses holistic, individualized care strategies, emphasizing the integration of patients’ life stories and preferences into the care process.

#### Barriers to person-centered car

While dedicated to implementing PCC, nurses identified significant barriers, including time constraints, resource limitations, and systemic challenges. Participant N2 expressed frustration over time constraints, “The biggest challenge is time. It’s difficult to practice true PCC when you’re seeing a high volume of patients with limited time for each.” This sentiment highlights the tension between the ideals of PCC and the realities of clinical practice. Participant N8 underscored systemic issues, “Organizational policies and resource shortages often impede our ability to offer personalized care.” These comments reveal the broader systemic and organizational obstacles to PCC implementation. Despite these challenges, Participant N5 shared, “We get creative, find small ways to personalize care within these constraints. It’s not easy, but it’s vital for maintaining the essence of PCC.”

### Experiences of caring for older adults

#### Building trust

In our study, nurses consistently highlighted the foundational importance of building trust in the nurse-patient relationship, especially when caring for older adults. This trust facilitates open communication, allowing patients to share their concerns, preferences, and life stories, which in turn informs personalized care planning. For example, Participant N2 shared a poignant experience: “Building trust with Mr. X allowed him to share his fears about aging and illness. It changed how we approached his care plan.” This sentiment was echoed by Participant N8, who emphasized the gradual nature of building trust: “Trust isn’t built overnight. It comes from consistent, compassionate care and really listening to what our patients value in their lives and health.” Further enriching this theme, Participant N6 remarked, “The moment a patient feels truly understood, that’s when real healing begins. Trust is that bridge.” The convergence of these experiences underscores the pivotal role of trust in enhancing nurse-patient interactions, leading to more effective and tailored care strategies.

#### Adapting care approaches

The necessity of flexibility and individualization in nursing care for older adults emerged as a critical theme. Our participants discussed the importance of adapting their care approaches to suit the diverse needs of older patients. Participant N4 described this adaptability as key: “Each patient is a unique puzzle. What works for one may not work for another. We constantly adapt our strategies to find what’s best for each individual.” Complementing this perspective, Participant N11 highlighted the necessity of adaptation in complex cases: “Adapting our approach is essential, especially when dealing with complex cases. It’s about finding the right balance that respects the patient’s lifestyle and preferences.” Participant N3 further illustrated this point by saying, “Listening and adjusting to the patient’s feedback is crucial. It’s about crafting care that aligns with their personal journey and health goals.” These narratives reflect the nurse’s role in continually assessing and modifying care plans, emphasizing personalized care that respects each patient’s individuality.

#### Interdisciplinary collaboration

Nurses also stressed the value of interdisciplinary collaboration in providing PCC care to older adults. This collaborative approach ensures that all aspects of a patient’s health are addressed. Participant N1 noted the importance of teamwork: “We work closely with dietitians, physiotherapists, and social workers. It’s a team effort to ensure our patients receive holistic care.” Participant N9 added, “Collaboration with other disciplines helps us address not just the physical, but also the emotional and social needs of our patients.” Expanding on this, Participant N7 shared, “Integrating insights from different fields brings a richness to the care plan that one discipline alone can’t provide.” This theme highlights how healthcare professionals can offer a more holistic approach by working together, addressing the physical, emotional, and social health of older patients.

#### Emotional rewards

Lastly, the emotional rewards of working with older adults were a theme that resonated deeply among the nurses we interviewed. Participant N5 expressed the profound satisfaction derived from their impact: “There’s a profound satisfaction in seeing the difference you can make in someone’s life, even in small ways.” Participant N10 reflected on the expressions of gratitude: “The thank yous, the smiles, even the silent gratitude you feel from a patient, it’s incredibly rewarding.” Additionally, Participant N12 captured the essence of this reward: “Knowing you’ve helped someone navigate one of the most challenging phases of their life is a privilege.” These reflections highlight the intrinsic satisfaction and fulfillment nurses experience, underscoring the significant impact of their work on the well-being and quality of life of their patients.

### Experiences in older adult care

#### Building Trust

Building trust is fundamental in the nurse-patient relationship, especially within the context of Person-Centered Care (PCC) for older adults. This trust fosters open communication, enabling patients to share their concerns, preferences, and life stories, which informs a more personalized care approach. For example, Participant N2 shared, “Building trust with Mr. X was transformative; his openness about his aging fears significantly informed our personalized care plan.” Participant N8 highlighted the gradual nature of trust-building: “Trust is cultivated through consistent, empathetic care, demonstrating genuine interest in the patient’s values and life.” Participant N6 further noted, “Feeling understood by a patient signifies the beginning of real healing. Trust serves as that crucial bridge.” These examples illustrate how trust facilitates effective PCC strategies, leading to tailored care solutions that respect the individuality of each older adult.

#### Adapting care approaches

Flexibility and individualization are essential in adapting care approaches under PCC. Nurses emphasized the importance of customizing care to meet the unique needs of each older adult. As Participant N4 noted, “Every patient presents a distinct puzzle,” underscoring the need for bespoke care strategies. Participant N11 discussed the complexity of adaptation: “Particularly with complex cases, finding a care balance that honors the patient’s lifestyle and preferences is paramount.” Participant N3 added, “Actively responding to patient feedback is key to aligning care with their personal health journey.” These insights highlight how adaptive strategies are crucial in developing care plans that are truly person-centered, reflecting each patient’s specific preferences and health goals.

### Interdisciplinary collaboration

Interdisciplinary collaboration plays a vital role in achieving comprehensive PCC by addressing the multifaceted needs of older adults. Nurses pointed out that teamwork across disciplines enhances the quality of care by integrating diverse professional insights. Participant N1 noted, “Collaboration across disciplines is fundamental,” indicating the collective effort required for holistic care. Participant N9 explained, “Working alongside dietitians, physiotherapists, and social workers allows us to meet the physical, emotional, and social health needs of our patients.” Participant N7 added, “The integration of diverse professional insights enriches the care plan, offering a multi-dimensional approach to patient well-being.” These perspectives demonstrate how collaborative practices support PCC, ensuring a holistic approach that covers all aspects of patient health and well-being.

### Emotional rewards

Engaging in PCC with older adults brings significant emotional rewards for nurses. The fulfillment from making a meaningful difference in a patient’s life is profoundly satisfying. As Participant N5 reflected, “The satisfaction from making a tangible difference in someone’s life is immense.” Participant N10 emphasized the personal impact of gratitude: “The appreciation, whether through words or smiles, is deeply rewarding.” Participant N12 summarized this sentiment, “Assisting someone through a challenging phase of their life is not just a duty but a privilege.” These reflections capture the intrinsic rewards of nursing, highlighting how PCC enhances the quality of life for older adults and provides personal and professional fulfillment for nurses.

### IV-Impact on Care Quality

#### Consistency in care

Achieving consistency in care within outpatient settings, amidst resourced constraints and emotional strain, is vital for embodying Person-Centered Care (PCC). Participants highlighted the challenges of fluctuating patient volumes and diverse health conditions impacting care uniformity. Participant N1 noted, “Striving for consistent care for every patient is our goal, but resource limitations often hinder this, impacting our ability to tailor care to individual patient preferences—a core aspect of PCC.” Participant N9 emphasized the importance of consistency to quality care and patient satisfaction, a sentiment echoed by Participant N2, who discussed the difficulty of balancing immediate needs with long-term care continuity, underscoring the effort to align care with each patient’s health priorities and personal preferences.

#### Patient satisfaction

The foundation of patient satisfaction in outpatient care is the quality of the nurse-patient relationship, integral to PCC. Participant N2 shared, “When I take the time to really listen to my patients and understand their concerns, they feel valued and are more satisfied with their care.” Similarly, Participant N5 noted, “Patients often tell me how much they appreciate being treated as individuals, not just another case. This personal connection makes a big difference in their satisfaction.” These insights underscore the importance of personalized, attentive care in enhancing patient satisfaction within the PCC framework. Participants described how personal attention and respect during each interaction significantly affect patients’ satisfaction. For example, Participant N5 said, “Being seen and heard elevates patient satisfaction, reflecting our commitment to understanding and incorporating their preferences and values into care.” Participant N11 added insights on building trust through respect, understanding, and care, crucial for patient-centered practice. Participant N4 highlighted how trust and rapport, established through PCC strategies like active listening and shared decision-making, encourage adherence to care plans.

#### Professional development

Nurses emphasized the crucial role of continuous learning in enhancing person-centered care (PCC). They shared specific examples illustrating how ongoing professional development translates into improved patient outcomes and more effective care strategies: Participant N3: Highlighted the impact of a recent workshop on geriatric communication strategies: “After attending a workshop on effective communication with older adults, I implemented techniques like using simpler language and allowing more time for responses. This change significantly improved my interactions with patients who have cognitive impairments, making them feel more understood and valued.” Participant N7: Described the application of new skills from a training program on chronic disease management: “The chronic disease management training I completed provided me with updated protocols for diabetes care. I started using patient-centered approaches to create personalized diabetes management plans, which led to better adherence to treatment regimens and improved glycemic control in my patients.” Participant N10: Discussed the benefits of learning about the latest advancements in PCC through continuing education: “Continuing education on the latest PCC advancements taught me about incorporating patients’ life histories into care planning. This knowledge helped me develop more holistic and individualized care plans, which have been particularly effective in managing complex cases involving multimorbidity.” These examples underscore the importance of professional growth in adapting care practices to reflect the latest PCC advancements, ensuring that nurses can effectively meet the diverse and complex needs of older adults in outpatient settings. Continuous learning equips nurses with the skills and knowledge to apply innovative PCC approaches, ultimately leading to improved patient satisfaction and care quality.

#### Ethical considerations

Ethical dilemmas are particularly prevalent in outpatient settings and require careful navigation to uphold Person-Centered Care (PCC) principles. These dilemmas often involve balancing patient autonomy, family wishes, and clinical guidelines to ensure high-quality care. N6 described a situation where an older patient with dementia wanted to continue living independently, despite safety concerns raised by family members. “We had to balance the patient’s wish for autonomy with the family’s concern for safety,” N6 explained. The ethical challenge was in respecting the patient’s desire while ensuring their safety, which was resolved through a compromise of enhanced home care support. Participant N8 recounted an instance where a patient with multiple chronic conditions refused a recommended treatment due to personal beliefs. “We faced the challenge of respecting the patient’s autonomy and belief system while trying to advocate for what we believed to be the best clinical course,” N8 noted. The team respected the patient’s decision after providing comprehensive information on the potential risks and benefits of the treatment, ensuring informed consent. Participant N12 highlighted an ethical dilemma involving a patient whose family insisted on aggressive treatment contrary to the patient’s expressed wishes for palliative care. “We navigated this by facilitating a family meeting where the patient’s preferences were clearly communicated and respected,” N12 shared. This approach helped align the care plan with the patient’s values while addressing family concerns through open dialogue.

### V-coping strategies supporting PCC

#### Peer support

The complexity of delivering PCC, which requires deep understanding and adaptation to each patient’s unique needs and preferences, heightens the value of peer support among nurses. Participant N2 highlighted the emotional relief provided by sharing experiences with colleagues: “Discussing challenging cases with a colleague helps us find new ways to approach patient care, ensuring we stay true to our PCC values.” Participant N8 noted the structured support from peer groups: “Our support groups focus on creative problem-solving in PCC, sharing strategies that respect patient autonomy and preferences.” This collaborative environment is crucial for nurses, allowing them to share PCC strategies and maintain emotional and professional resilience.

#### Reflective practice

Reflection is a powerful tool for nurses, enabling them to assess and refine their approach to PCC. Participant N1 described using reflection to enhance patient interactions: “Reflecting on patient feedback has helped me better understand their preferences, which is central to PCC.” Participant N9 uses journaling to process experiences, aiding in the development of more empathetic and patient-centered care strategies.

#### Resilience building

The demands of delivering PCC in outpatient settings necessitate resilience. Participant N3 found mindfulness meditation helpful for maintaining focus on patient needs, even in stressful situations. Participant N7 shared the benefits of resilience workshops: “Learning stress management techniques has improved my ability to adapt care plans according to patient preferences, a key aspect of PCC.” This focus on resilience supports nurses in consistently applying PCC principles, even when faced with challenges.

## Discussion

This qualitative study offers invaluable insights into the intricate dynamics and challenges that shape the nurse-patient relationship within the context of caring for older adults in outpatient settings. By amplifying the voices of experienced nurses, our findings illuminate the multifaceted nature of delivering person-centered care (PCC) to this vulnerable patient population. The thematic analysis sheds light on the lived experiences of nurses, the obstacles they confront, and the strategies they employ to navigate these complexities—all while striving to uphold the principles of PCC and address the diverse health priorities of older adults.

In our exploration of Person-Centered Care (PCC) in outpatient settings, this study illuminates the nuanced ways in which nurses navigate the delicate balance between addressing health priorities and enhancing the quality of life for older adults. While the focus on managing chronic conditions and immediate health concerns is paramount, our findings underscore the equally critical endeavor of enhancing patients’ holistic well-being. Nurses, through their application of PCC principles, actively consider the broader aspects of older adults’ lives, integrating concerns related to emotional, social, and spiritual well-being into their care plans.

Moreover, the findings reveal that the strategies employed by nurses to adapt care approaches and engage in interdisciplinary collaboration have profound implications for patients’ quality of life. By tailoring care to reflect each individual’s preferences and involving a spectrum of healthcare professionals, nurses ensure that care plans are both comprehensive and conducive to enhancing well-being beyond the clinical domain [43]. These insights enrich the discourse on PCC by demonstrating that the concept extends far beyond the confines of individualized medical treatment. Rather, PCC embodies a holistic approach that considers the entirety of the patient’s life context, emphasizing the importance of quality of life as a pivotal component of care [44].

The significance of trust as a foundational element in the nurse-patient relationship emerges as a resounding theme. Nurses emphasize the paramount importance of building trust, recognizing it as a catalyst for effective communication, collaborative care planning, and ultimately, improved health outcomes. This finding resonates with existing literature that highlights trust as a crucial determinant of patient satisfaction, adherence to treatment regimens, and overall quality of care [45–47]. Notably, the process of trust-building is an ongoing endeavor, requiring consistent, empathetic, and patient-centered interactions, as emphasized by participants in our study [48]. This underscores the need for healthcare systems and institutions to foster environments that promote and value the nurturing of nurse-patient trust [49].

The subtheme of adapting care approaches emerges as a central tenet of PCC in the care of older adults. Nurses acknowledge the inherent diversity among this patient population, acknowledging that a one-size-fits-all approach is inadequate in addressing the complexities of aging and associated health challenges. This finding aligns with the principles of PCC, which emphasize the importance of tailoring care to individual needs, preferences, and values [50]. By continually adapting their strategies, nurses strive to provide care that respects the uniqueness of each older adult, ultimately enhancing patient outcomes and satisfaction [51].

Interdisciplinary collaboration arises as a pivotal theme, reflecting the multifaceted nature of caring for older adults [52]. Nurses recognize the invaluable contributions of diverse healthcare professionals, including dietitians, physiotherapists, social workers, and mental health specialists, in addressing the physical, emotional, and social dimensions of health [53]. This finding resonates with the growing body of literature advocating for interdisciplinary care models as a means to provide comprehensive, holistic support for older adults [54]. By embracing a collaborative approach, nurses can leverage the collective expertise of the healthcare team, ensuring that care plans encompass the full spectrum of an older adult’s health priorities [55].

The emotional rewards of caring for older adults emerge as a poignant and motivating aspect of the nurse-patient relationship. Nurses derive profound satisfaction and fulfillment from witnessing the positive impact of their care, be it through explicit expressions of gratitude or the subtle yet palpable sense of appreciation from patients and their families [56]. This finding aligns with previous studies that have explored the intrinsic rewards of nursing, particularly in the context of geriatric care [57]. The emotional rewards documented in our study serve as a powerful reminder of the profound significance of nursing’s role in improving the quality of life for older adults, reinforcing the value and meaning inherent in this profession [58].

Our study makes several significant contributions to the literature on Person-Centered Care (PCC) in outpatient settings, particularly in the context of nursing care for older adults. While existing research has extensively explored PCC within inpatient settings, our work extends this discourse by illuminating the unique challenges and strategies inherent in outpatient care. One of the key contributions of this study is the detailed exploration of how nurses navigate the complexities of implementing PCC with limited resources and within the constraints of outpatient environments. Unlike previous studies that primarily focus on the conceptualization and benefits of PCC, our findings offer a pragmatic look into the adaptive strategies nurses employ to maintain PCC principles despite these challenges.

Furthermore, this study contributes to the literature by providing insights into the specific coping mechanisms nurses utilize to sustain their well-being and professional satisfaction while delivering PCC. This aspect of our research underscores the critical link between nurse well-being and the quality of patient care, highlighting the importance of supporting nursing staff in their roles. Additionally, our work sheds light on the nuanced ways in which nurses build trust and engage in reflective practices to enhance the delivery of PCC, offering concrete examples that can inform training and development programs aimed at bolstering PCC in outpatient settings.

### Limitations of the study

This qualitative study provides insights into the nurse-patient relationship for older adults in outpatient settings but has several limitations. Conducted in a specific geographic region, the findings might not apply universally due to varying cultural and healthcare contexts. The focus on nurses’ perspectives, excluding those of older adults and their families, may limit the comprehensiveness of the results. The small sample size of 12 nurses may not fully represent diverse nursing experiences, and reliance on self-reported data could introduce bias. Additionally, its cross-sectional design captures only a temporal snapshot of the dynamics within nurse-patient interactions, limiting the understanding of their evolution over time.

### Implications of the study

The study’s findings emphasize the need for trust-building, adaptive care strategies, and interdisciplinary collaboration in nursing practice. Healthcare organizations should foster environments that support effective person-centered care and professional development for nurses. Educational programs must enhance training in communication, ethical decision-making, and family dynamics management. Policymakers should ensure adequate resources and support for nurses to improve care for the growing population of older adults. Future research should expand on these findings with longitudinal studies and broader participant perspectives to develop targeted interventions that enhance both nurse-patient relationships and overall care quality.

### Recommendations

Based on our findings, several concrete recommendations can be made for practice, research, and education in the field of nursing, particularly within the context of Person-Centered Care (PCC) for older adults in outpatient settings.

#### Practice

Our study underscores the importance of continuous professional development in PCC practices tailored specifically to outpatient care. Healthcare institutions should consider implementing regular training sessions that focus on enhancing communication skills, empathy, and the understanding of the unique needs of older adults. Additionally, developing policies that encourage more time for nurse-patient interactions can improve the implementation of PCC, leading to better patient outcomes.

#### Research

Future research should explore the implementation of PCC in varied healthcare settings to compare how different environments influence the effectiveness of these practices. Investigating the applicability of our findings in rural versus urban settings, or in specialized settings such as dementia care, could provide deeper insights into the adaptability and scalability of PCC strategies.

#### Education

Educational curricula for nursing students should incorporate modules that emphasize the theoretical and practical aspects of PCC. This could include case studies drawn from real-world settings that illustrate both successful and challenging aspects of applying PCC principles. Additionally, simulation-based training could be used to prepare students to handle complex situations involving older adults, enhancing their readiness for real-world challenges.

#### Transferability of findings

While the findings from our study are grounded in the context of outpatient clinics, the insights regarding the dynamics of nurse-patient relationships and the challenges of implementing PCC have potential applicability to other settings. However, it is important to consider the specific cultural, organizational, and resource-based differences that might affect the transferability of these practices. Researchers and practitioners should carefully evaluate the context-specific factors in their own settings before adopting the recommendations from this study.

By addressing these recommendations, we can enhance the quality of nursing practice, enrich the educational experiences of nursing students, and guide future research towards areas that will maximize the impact of PCC across diverse healthcare environments.

## Conclusion

This qualitative study provides key insights into the nurse-patient relationship and the delivery of person-centered care to older adults in outpatient settings. It highlights the importance of trust-building, effective communication, and adaptive care strategies tailored to the diverse needs of older adults. The research emphasizes the crucial role of interdisciplinary collaboration in managing complex health priorities and underscores the challenges nurses face, such as resource constraints, family dynamics, emotional strain, and the need for continuous professional development.

The study reveals that coping strategies like peer support, maintaining work-life balance, engaging in reflective practice, and resilience-building are vital for enhancing both nurse well-being and the quality of care. These findings have significant implications for nursing practice, education, policy, and research, urging healthcare organizations to create environments that support strong nurse-patient relationships and comprehensive, person-centered care.

Despite its limitations, including geographic specificity and reliance on self-reported data, the study contributes valuable perspectives to the understanding of caregiving in geriatric outpatient settings. As the population of older adults grows, the role of nurses in providing person-centered care is increasingly essential. This study calls for healthcare systems to acknowledge and support nurses’ critical contributions, ensuring that the aging population receives the compassionate, individualized care they need to improve their quality of life.

### Electronic supplementary material

Below is the link to the electronic supplementary material.

Supplementary Material 1

Supplementary Material 2

## Data Availability

The datasets generated during and/or analyzed during the current study are available from the corresponding author on reasonable request.
