# Supplementary material for: Exploring the nurse-patient relationship in caring for the health priorities of older adults: qualitative study
Source: BMC Nurs. 2024 Jul 15;23:480. doi: 10.1186/s12912-024-02099-1 (PMC11247866; doi:10.1186/s12912-024-02099-1)
Supplement: Supplementary file 1 — Supplementary Material 1 [file 12912_2024_2099_MOESM1_ESM.docx]

**Table S1: Interview Questions on Experiences and PCC in Outpatient Settings**

| **Question Number** | **Interview Question** |
| --- | --- |
| Q1 | Can you describe a particularly memorable experience you've had with an older adult in the outpatient clinic that reflects the principles of Person-Centered Care? |
| Q2 | How do you tailor your communication and care strategies to align with the individual preferences and needs of your older adult patients? |
| Q3 | Reflecting on your time in the outpatient clinic, what challenges have you encountered in implementing PCC with older adults, and how have you addressed these challenges? |
| Q4 | Can you share an example of how interdisciplinary collaboration in the outpatient clinic has enhanced the delivery of PCC to an older adult? |
| Q5 | In what ways has working with older adults in an outpatient setting influenced your perspective on nursing care and the application of PCC principles? |
