# Supplementary material for: Exploring the nurse-patient relationship in caring for the health priorities of older adults: qualitative study
Source: BMC Nurs. 2024 Jul 15;23:480. doi: 10.1186/s12912-024-02099-1 (PMC11247866; doi:10.1186/s12912-024-02099-1)
Supplement: Supplementary file 2 — Supplementary Material 2 [file 12912_2024_2099_MOESM2_ESM.docx]

**supplementary table 2. Thematic Results: Experiences, Challenges, and Strategies in Nurse-Patient Relationships for Older Adult Care**

| **Theme** | **Subtheme** | **Description** |
| --- | --- | --- |
| **Understanding and Implementing Person-Centered Care (PCC)** | **Defining Person-Centered Care** | Nurses describe PCC as a holistic approach that prioritizes the patient's preferences, values, and needs, ensuring that care is tailored to the individual |
|  | **Barriers to Person-Centered Care** | Common obstacles include time constraints due to high patient loads, limited resources that prevent personalized care delivery, and organizational policies that do not always support flexible, patient-centered approaches |
| **Experiences in Older Adult Care** | **Building Trust** | Nurses highlight the importance of building trust with older adults, noting it as fundamental for effective communication and care planning. |
|  | **Adapting Care Approaches** | Participants discuss the need for adapting care strategies to meet the diverse needs of older adults, emphasizing personalized care based on individual health priorities. |
|  | **Interdisciplinary Collaboration** | Nurses underscore the value of working within interdisciplinary teams to address complex health needs, ensuring comprehensive care. |
|  | **Emotional Rewards** | Sharing experiences of gratitude and emotional fulfillment from patients and families, nurses describe these interactions as highly rewarding aspects of their work. |
| **Challenges in Care Delivery** | **Resource Constraints** | Nurses’ express concerns over limited resources and staffing, which impact their ability to provide person-centered care consistently. |
|  | **Navigating Family Dynamics** | Challenges in dealing with complex family dynamics and expectations in the care of older adults are discussed, highlighting the need for effective communication skills. |
|  | **Keeping Up with Medical Advances** | Staying informed on the latest in geriatric care and integrating new practices into patient care pose challenges due to the rapid pace of medical advancements. |
|  | **Emotional Strain** | The emotional toll of caring for older adults, especially those nearing end-of-life, is highlighted, with nurses discussing the impact on their mental health. |
| **Impact on Care Quality** | **Consistency in Care** | Nurses point out that challenges like resource constraints and emotional strain can lead to inconsistencies in the quality of care provided to older adults. |
|  | **Patient Satisfaction** | The role of nurse-patient relationships in enhancing patient satisfaction is emphasized, with a focus on how personalized, compassionate care leads to better patient outcomes. |
|  | **Professional Development** | Continuous professional development is identified as key to maintaining high standards of care, with nurses seeking opportunities for training and growth. |
|  | **Ethical Considerations** | Navigating ethical dilemmas, especially concerning autonomy and informed consent in older adult care, is discussed as a critical aspect impacting care quality. |
| **Coping Strategies** | **Peer Support** | The importance of peer support and team debriefings as strategies to manage the emotional and professional challenges of caring for older adults is highlighted. |
|  | **Reflective Practice** | Engaging in reflective practice is discussed as a method for personal and professional growth, helping nurses to continuously improve their approach to patient care. |
|  | **Resilience Building** | Strategies for building resilience, including mindfulness and stress management techniques, are considered essential for coping with the demands of caring for older adults in outpatient settings. |
